# Trends in Ambulatory Analgesic Usage after Myocardial Infarction: A Nationwide Cross-Sectional Study of Real-World Data

**DOI:** 10.3390/healthcare10030446

**Published:** 2022-02-26

**Authors:** Sun-Young Jung, Seung Yeon Song, Eunyoung Kim

**Affiliations:** 1 College of Pharmacy, Chung-Ang University, Seoul 06974, Korea; jsyoung@cau.ac.kr; 2 Data Science, Evidence-Based and Clinical Research Laboratory, Department of Health, Social and Clinical Pharmacy, College of Pharmacy, Chung-Ang University, Seoul 06974, Korea; tmddus0121@hotmail.com; 3 Department of Pharmaceutical Industry, Graduate School, College of Pharmacy, Chung-Ang University, Seoul 06974, Korea

**Keywords:** myocardial infarction, non-steroidal anti-inflammatory drugs (NSAIDs), the Korea National Health Insurance Service (NHIS) database, ambulatory analgesics, patterns and trends

## Abstract

Although current guidelines for myocardial infarction (MI) recommend caution in using non-steroidal anti-inflammatory drugs (NSAIDs), real-world studies of ambulatory settings are rare. This study aimed to explore the patterns and trends of analgesic prescriptions (especially NSAIDs) among patients with a history of MI in ambulatory care settings in Korea. We analyzed real-world data from the Korea National Health Insurance Service database. Patients aged 20 years or older hospitalized with incident MI were identified between January 2007 and December 2015. Ambulatory analgesics were administered after discharge from incident hospitalization for MI, and annual trends in the prescriptions of individual analgesics were evaluated. Among the 93,597 patients with incident MI, 75,131 (80.3%) received a total of 2,081,705 ambulatory analgesic prescriptions. Prescriptions were mainly issued at primary care clinics (80.3%). Analgesics were most frequently prescribed for musculoskeletal diseases (often NSAIDs, 70.7%); aceclofenac (13.7%) and diclofenac injection (9.4%) were the frequently used NSAIDs. Additionally, significant changes were observed in the trends for some analgesics, such as loxoprofen. This study suggested that NSAIDs are commonly prescribed to patients with a history of MI. Future real-world studies are needed to elucidate the drug–disease interactions of NSAIDs prescribed after MI, especially for patients with musculoskeletal diseases.

## 1. Introduction

Myocardial infarction (MI) is a leading cause of premature death and represents a major disease burden, with the growing proportion of aged individuals globally [1,2]. Although several patients who experience an MI survive, many of the survivors may experience subsequent cardiovascular events, such as a stroke, another MI, or cardiovascular death [3]. Analgesics, including acetaminophen and non-steroidal anti-inflammatory drugs (NSAIDs), are commonly used for the symptomatic treatment of comorbid conditions causing pain, fever, and inflammation [4,5]. However, concerns regarding the cardiovascular safety of NSAIDs, particularly cyclooxygenase-2 (COX-2) inhibitors and diclofenac, are reported [6,7,8]. Therefore, the current guidelines for MI recommend caution when using COX-2-selective or non-selective NSAIDs [9,10]. Although several studies have identified risk factors for safety outcomes associated with NSAIDs [11,12,13], very few have investigated the patterns of ambulatory analgesic prescriptions in real-world practice [3,11].

A recent study using the Korean nationwide real-world prescription claims database found that concomitant NSAID treatment promoted significantly greater risk for cardiovascular and bleeding events than no NSAID treatment [14]. Considering the diverse comorbidities that can occur after MI, patients may visit physicians who specialize in fields other than cardiovascular medicine, substantially increasing the possibility of analgesic prescriptions without considering the patient’s history of MI. Therefore, drug-utilization studies using real-world data that focus on the major diagnoses leading to analgesic prescriptions and trends in analgesic use while considering the cardiovascular safety of NSAIDs after MI are needed to promote proper drug use.

To explore the trends and patterns of analgesic prescriptions, particularly NSAIDs, in patients who had suffered an MI in ambulatory care settings, this study aimed to analyze nationwide real-world data obtained over an 8-year period and determine trends in the most common major indications.

## 2. Materials and Methods

### 2.1. Data Source and Study Population

Data from the Korean National Health Insurance Service (NHIS) database between January 2007 and December 2015 were extracted [15]. In Korea, a mandatory universal health insurance program provides comprehensive medical care coverage to 97% of the population. The Medical Aid program, instituted for the low-income population, covers the remaining 3%. Information from both universal health insurance and the Medical Aid program are recorded within a single NHIS database. The NHIS database contains information on patient demographics, health care use, and prescribed drugs for approximately 50 million Korean citizens. The database uses anonymized patient codes, diagnoses based on the International Classification of Disease (ICD-10) codes, visitation dates, and prescription and procedure history. This study was approved by the Chung-Ang University Bioethics Committee (No 1041078-201603-HR-066-01) and NHIS.

**Figure 1:**
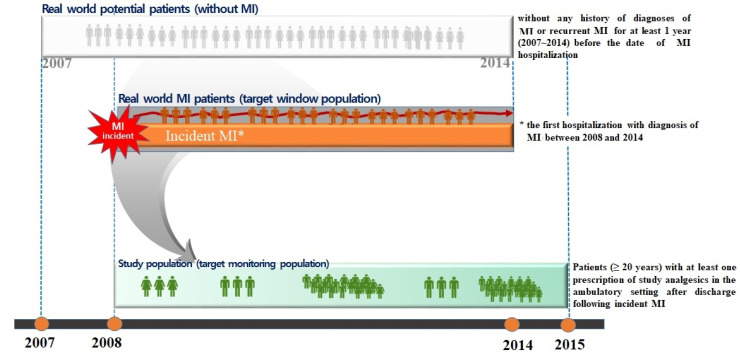
Graphical depiction of the study design.

The whole data set was extracted from 2007 to 2015. The study population consisted of patients aged 20 years or older with at least one prescription of the study analgesics in an ambulatory care setting after discharge following incident MI. For this study, incident MI was defined as the first hospitalization with a diagnosis of MI between 2008 and 2014 without any history of MI diagnosis or recurrent MI for at least 1 year before the date of MI hospitalization (Figure 1).

### 2.2. Assessment of Analgesic Use

Analgesics assessed herein included acetaminophen (WHO ATC code, N02BE01), opioids (N02A), salicylic acid and derivatives (N02BA), and non-steroidal anti-inflammatory products (M01A). Only prescriptions dated at least 30 days after discharge for incident MI were included to exclude analgesics used for in-hospital or postoperative care. The use of oral medications, injections, and topical agents was studied separately to determine actual patterns in analgesic usage. For instance, tramadol and tramadol injections were counted as separate medications.

Patterns of analgesic prescription were based on the corresponding ICD-10 diagnoses. The following patient and prescription characteristics for cases in which analgesics were prescribed after MI were assessed: age, sex, type of insurance, type of medical institution (tertiary hospital, general hospital, hospital, primary clinic, and public health center), prescriber’s medical specialty, and comorbidities (heart failure, arrhythmia, cerebrovascular disease, dyslipidemia, peptic ulcer disease, peripheral vascular disease, renal failure, hypertension, diabetes mellitus, and cancer) [16].

### 2.3. Statistical Analysis

This study used descriptive statistics to assess the baseline characteristics of the study population and the overall analgesic-containing prescriptions. During person-based analysis, the presence of comorbidities was defined based on the presence of one or more diagnoses during the study period. Moreover, the type of medical institution was defined based on the institution type most frequently visited during the study period.

The prevalence of analgesic use in ambulatory care settings within the 8-year study period was estimated. For prescriptions including analgesics as the unit of analysis, the Chi-square test was used to compare the proportion of prescriptions with NSAIDs for each indication. For further prescription-based analysis, trends in analgesic combinations for the same prescription between 2008 and 2015 were assessed according to the primary diagnosis in each prescription. Indications of analgesic use were defined using the primary diagnosis of each prescription containing analgesics. Analyses were also performed using the individual analgesic medication as the unit of analysis. The 20 most commonly prescribed individual analgesic medications in patients with a history of MI were compared according to the primary diagnosis of each prescription. Moreover, time-series analysis was performed for individual analgesics. All analyses were computed using SAS version 9.4 (SAS Institute, Cary, NC, USA).

## 3. Results

Between January 2008 and December 2014, 93,597 patients aged 20 years or older were hospitalized for incident MI. After applying the additional inclusion criterion of a prescription for the study analgesics at least 30 days after discharge, the final sample consisted of 75,131 patients (80.3% of the patients with incident MI) who had received 2,081,705 ambulatory analgesic prescriptions.

**Figure 2:**
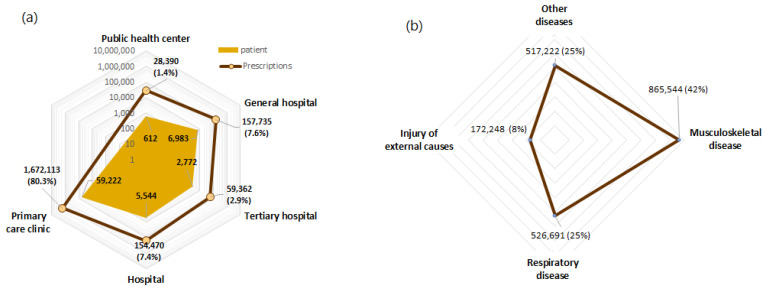
Trends and patterns of analgesic prescriptions with NSAIDs in ambulatory care settings among patients with a history of MI: (**a**) Type of medical institution and the number of analgesic prescriptions/patients. (**b**) The proportion of prescriptions with NSAIDs for each indication among patients with incident MI (2008–2015).

The characteristics of the study patients and prescriptions are presented in Table 1. The most prevalent comorbidities were dyslipidemia, hypertension, and diabetes mellitus in 97.2%, 95.5%, and 69.2% of the patients, respectively. Ambulatory prescriptions of analgesics were issued mainly at primary care clinics (80.3%), followed by general hospitals (7.6%), and hospitals (7.4%) (Figure 2a).

After categorizing prescriptions based on the prescriber’s specialty, our results showed that 34.5% were issued by an orthopedic specialist, whereas 32.3% were issued by an internal medicine specialist. Given that analgesics and NSAIDs are prescribed for various conditions, the top three diagnoses for which analgesics were prescribed (musculoskeletal diseases, respiratory diseases, and injuries attributable to external causes, among other diseases) were selected [17,18] (Figure 2b). The mean number of analgesic prescriptions per person for musculoskeletal diseases, respiratory diseases, and injuries of external causes in patients with incident MI was 11.52, 7.01, and 2.30, respectively. Moreover, the mean number of individual analgesics per prescription for each of these primary diagnoses was 1.41%, 1.35%, and 1.29%, respectively.

**Figure 3:**
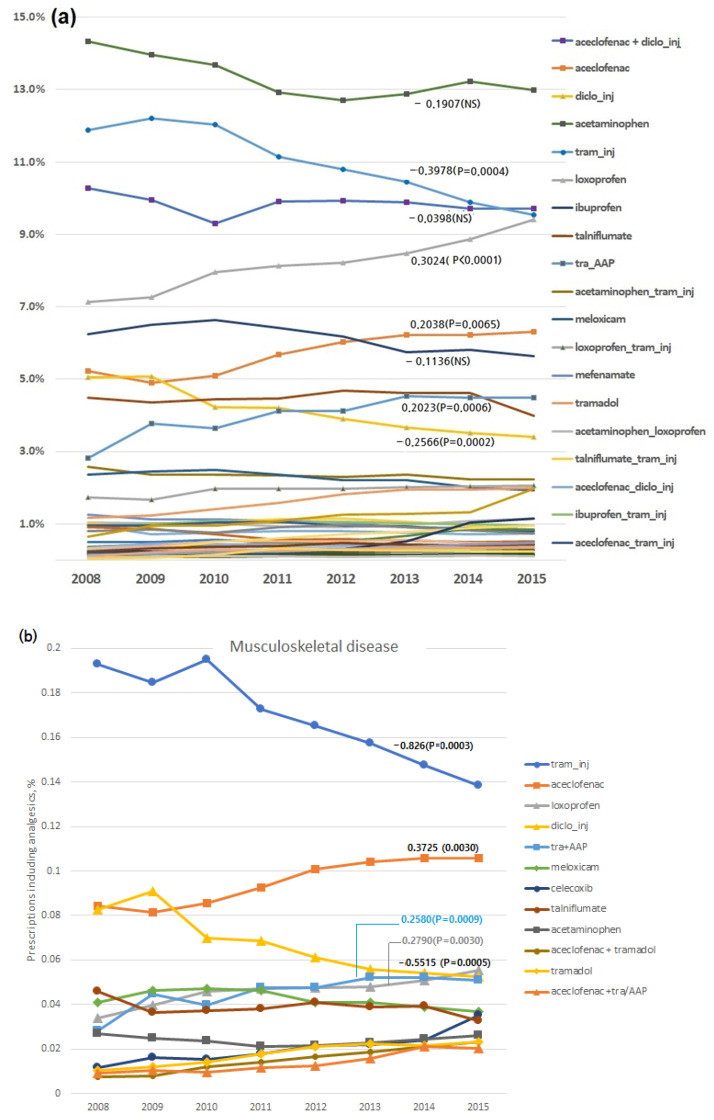
Time trends in the proportion of prescribed analgesics among patients with a history of MI during study years and time trend analysis (trends’ significance was expressed as beta value and *p*-value): (**a**) Overall trends in individual analgesics and (**b**) trends in musculoskeletal diseases (left: the proportions of the prescribed analgesics, right: time trends of individual analgesics). AAP: Acetaminophen, NS: Not Significant, tram: tramadol.

The three most commonly prescribed analgesics to patients who had MI with a primary diagnosis of musculoskeletal disease included tramadol injection (19.4%), aceclofenac (13.7%), and diclofenac injection (9.4%). The overall trends for individual analgesics over the study period are shown in Figure 3a. Notably, the trends showed significant reductions in prescriptions of tramadol injections (trend value, 0.3978; 18.1% ➔ 15.95%) and diclofenac injection (0.2566; 8.2% ➔ 6.5%) and significant increases in the use of loxoprofen (0.3024; 9.3% ➔ 11.8%), aceclofenac (0.2037; 7.4% ➔ 8.8%), and the tramadol + acetaminophen fixed combination (0.2023; 4.5% ➔ 7.1%). For musculoskeletal diseases (Figure 3b), the trends indicated significant reductions in prescriptions of tramadol (trend value, −0.826; 19.3% ➔ 13.8%) and diclofenac injections (−0.5515; 8.3% ➔ 5.3%) and significant increases in prescriptions of aceclofenac (0.3725; 8.4% ➔ 10.6%), loxoprofen (0.2790; 3.4% ➔ 5.6%), and the tramadol/acetaminophen fixed combination (0.2580; 2.8% ➔ 5.1%).

**Figure 4:**
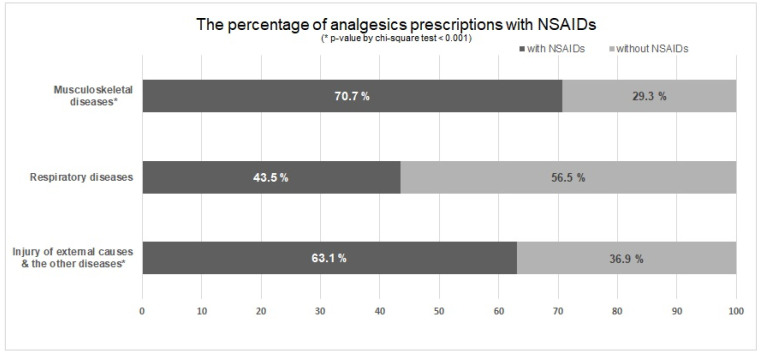
The proportions of non-steroidal anti-inflammatory drug (NSAID) prescriptions in analgesics for each indication among patients with a history of myocardial infarction.

For respiratory diseases and other diseases, the proportions of prescriptions of each analgesic and their time trends are shown in Supplementary Figure S1. The proportions of NSAID prescriptions for each indication among patients with a history of MI during this study period are presented in Figure 4. Accordingly, 43.5% of prescriptions for respiratory diseases included NSAIDs, whereas 70.7% of prescriptions for musculoskeletal diseases included NSAIDs (*p*-value by Chi-square test < 0.001).

## 4. Discussion

The current population-based analysis of adults with a history of MI found that analgesic prescriptions differed according to each indication for analgesic use. Although tramadol injection, aceclofenac, and diclofenac injection were most frequently used for musculoskeletal diseases, non-NSAIDs (e.g., acetaminophen and tramadol) and loxoprofen (a mixed COX-1/COX-2 inhibitor) constituted more than 50% of the overall analgesics prescribed for respiratory and other diseases.

The current guidelines on MI recommend a stepped-care approach to analgesic therapy. Initial therapy with acetaminophen, small doses of narcotics, or nonacetylated salicylates are recommended when introducing non-selective NSAIDs, which can be followed by increasing the degree of relative COX-2 selectivity, with the lowest effective doses administered for the shortest possible time [9,10]. Two major COX isoenzymes, COX-1 and COX-2, are involved in the production of prostaglandins from arachidonic acid. NSAIDs inhibit the COX enzymes. Platelets play an important role in cardiovascular haemostasis. Platelets express only COX-1 and produce thromboxane A2, which stimulates platelet aggregation and vasoconstriction, and increases vascular and cardiac remodelling. A potential pathology for the cardiac harm of NSAIDs is the observed shift in the prothrombotic/antithrombotic balance on endothelial surfaces towards thrombosis after NSAID exposure [10]. However, four-fifths of the patients with incident MI included herein received ambulatory analgesic prescriptions. Over 80% of the prescriptions were issued at primary care clinics, with most patients having cardiovascular comorbidities, such as dyslipidemia, hypertension, or heart failure. These findings warrant further investigation into the safety of analgesics in patients with a history of MI.

This study found a difference in the degree of NSAID use between musculoskeletal and respiratory diseases. This may be attributed to the clinical practice guidelines for each disease, with some guidelines considering NSAIDs superior to acetaminophen for the treatment of osteoarthritis [19]. For respiratory diseases, acetaminophen and ibuprofen are frequently prescribed as antipyretics. A previous study in India also reported a higher prevalence of diclofenac or piroxicam prescriptions by orthopedic specialists [17]. Alternatively, this may reflect the possibility that the extent of information recorded regarding the patient’s history of MI differs between departments and physician specialties. In fact, a previous study performed in the European Union revealed that patients with comorbidities under the care of different specialists were reported being at increased risk for adverse drug events [20]. Thus, efforts are required to address the insufficient consideration of comorbidities when prescribing drugs. One solution would be the adoption of nationwide information tools that provide potential drug-disease interaction data to physicians.

Although diclofenac remains a highly prescribed drug, the number of diclofenac prescriptions has decreased over time. This trend may reflect continued concerns regarding increased risk for cardiovascular complications with COX-2-selective inhibitors, which was first proposed in the early 2000s [7]. Since 2011, meta-analyses have reported that the risk for cardiovascular events, mainly MI, associated with high doses of diclofenac was comparable to that associated with COX-2 selective inhibitors [6,8]. A study in the US also reported a reduction in the postoperative use of NSAIDs after coronary artery bypass graft surgery between 2004 and 2010, especially after the black box warning of cardiovascular risk [21]. During the study period, the proportion of celecoxib prescriptions in our population was low but increased slightly (0.9% ➔ 2.2%). This can be explained by the difference in profile between celecoxib and rofecoxib despite both being classified as COX-2-selective inhibitors [8,10,11,14]. Moreover, a recent study using the Korean NHIS database considered celecoxib as an alternative option in cases in which NSAID use was unavoidable [14].

In contrast, our findings showed that the use of aceclofenac and loxoprofen increased significantly (Figure 3a). Aceclofenac was the most frequently prescribed oral analgesic for musculoskeletal disease (Figure 3b). However, a recent Italian study reported that only 7.5% and 1.3% of patients with cerebral/cardiovascular disease were prescribed diclofenac and aceclofenac for musculoskeletal indications, respectively [22]. Nevertheless, more data are required to determine whether aceclofenac was prescribed as a designated substitute for diclofenac. Aceclofenac is a prodrug developed in Spain [23] and is metabolized into 4′-hydroxyaceclofenac and diclofenac after oral ingestion [24]. Despite the continued reporting of cardiovascular risk associated with diclofenac [25], the prescription rate of aceclofenac has increased. Moreover, studies comparing the cardiovascular safety of aceclofenac and diclofenac are infrequent, although a trial involving 120 patients with osteoarthritis reported a better safety profile for aceclofenac [26]. A population-based case–control study conducted in Finland included aceclofenac as a study drug, although it was categorized under “other drugs” given the small number of exposures, for which its odds ratio was not calculated [27].

Loxoprofen is another popular NSAID in Korea and Japan. However, it is considered a prodrug-type NSAID with relatively weak gastrointestinal (GI) ulcerogenicity [28,29]. A population-based case–control study performed in Japan reported an increased risk of upper GI bleeding with the use of loxoprofen [30]. Considering the lack of studies on the cardiovascular safety of loxoprofen, additional population-level research examining the safety profile of aceclofenac and loxoprofen is required in countries where both drugs have been approved.

The term “balloon effect” is often used in drug policy to describe the phenomenon where problems are displaced rather than being truly solved, such as when a latex balloon is squeezed, the squeezed area shrinks, and the other part of the balloon expands. The increasing trends in aceclofenac and loxoprofen prescriptions can be attributed to a potential “balloon effect” for the need to avoid diclofenac or other NSAIDs due to safety concerns. Although both NSAIDs are commonly used in Asian countries, they are less common in the European Union or US. Given that most published studies were conducted in Western countries, the cardiovascular safety of both analgesics was not assessed and was consequently overlooked. This balloon effect supports the need for nationwide strengthening of and international collaboration on safety monitoring and safety evaluation of locally popular prescription drugs for which safety evidence is lacking. In particular, the relevant regulatory agencies should pay attention to these aspects to manage systematic and comprehensive regulations on safety.

Another notable result is the considerable use of injections in outpatient settings. Despite their decreasing trend, tramadol and aceclofenac injections were ranked first and fourth in the overall outpatient medication prescriptions. The overuse of injections in outpatient visits based on prescribers’ overconfidence and patients’ lack of knowledge [31] can be attributed to the cultural background of traditional medicine involving acupuncture and has continued to be an issue in Korea. A previous Korean study reported a two-fold higher use of injections in musculoskeletal diseases compared to other types of medications [32], similar to that shown in our analysis. The decreasing trend in the use of injections appears to be due to the rate monitoring and disclosure policy for outpatient injection prescriptions since 2007. According to a recent national report, the prescription rate of injections in overall outpatient visits throughout Korea decreased from 24.4% in 2008 to 17.6% in 2017 [33,34]. Our results indicate that further improvement is needed to improve the knowledge and perceptions regarding irrational injection use in outpatient visits.

Although current guidelines recommend avoiding NSAID or COX-2 inhibitor usage in patients with a history of MI [9,10], real-world evidence regarding the status of general analgesic usage remains scarce. This population-based study examined trends in the prescriptions of analgesics in patients with a history of MI using a database including the entire Korean population. However, the results should be interpreted after considering some of the limitations associated with the nature of claims databases. The NHIS database includes only data on reimbursed treatments administered in medical institutions or drugs dispensed from prescriptions. As such, data for over-the-counter drugs were not captured. The selection of analgesics by health care professionals informed on the patient’s history of MI and risk of NSAIDs may significantly impact the use of analgesics in our study population. Therefore, this study focused on prescription analgesics. Moreover, given that this study only assessed information from patients with a history of MI included in the database, the prescription patterns obtained herein cannot be extrapolated to patients without a history of MI. Therefore, our study did not examine factors associated with NSAID use after MI, which warrants further investigation. The key differences in the administration of medication between medical institutions should also be included in future studies.

In summary, the current study determined the general patterns of analgesic use for each indication studied among adult patients with a history of MI. The importance of adequate knowledge regarding the drug-disease interactions of NSAIDs used after MI needs to be further highlighted, especially for patients with musculoskeletal diseases. The high prevalence of the prescription of aceclofenac, loxoprofen, and injectable analgesics in ambulatory care was notable in this population.

## Figures and Tables

**Table 1:** General characteristics of prescriptions and patients with analgesic prescriptions who had a history of myocardial infarction between 2008 and 2015.

	Patients (*n*, %)		Prescriptions (*n*, %)	
Total	75,131	100%	2,081,705	100%
Sex				
Male	54,942	73.1	1,290,003	62.0
Female	20,189	26.9	791,702	38.0
Age (years)				
20–29	196	0.3	1558	0.1
30–39	2126	2.8	21,591	1.0
40–49	10,293	13.7	132,499	6.4
50–59	19,652	26.2	361,434	17.4
60–69	18,806	25.0	552,252	26.5
70–79	17,056	22.7	735,109	35.3
80+	7002	9.3	277,262	13.3
Type of insurance				
National health insurance	67,659	90.1	1,785,793	85.8
Medical aid	7472	9.9	295,912	14.2
Comorbidity				
Heart failure	39,982	53.2	1,164,319	55.9
Arrhythmia	18,037	24.0	580,302	27.9
Cerebrovascular disease	24,849	33.1	885,920	42.6
Dyslipidemia	73,025	97.2	2,035,744	97.8
Peptic ulcer disease	52,664	70.1	1,715,287	82.4
Peripheral vascular disease	35,541	47.3	1,288,322	61.9
Renal failure	6929	9.2	215,872	10.4
Hypertension	71,710	95.5	2,022,066	97.1
Diabetes mellitus	51,965	69.2	1,557,393	74.8
Cancer	12,148	16.2	409,877	19.7

## Data Availability

All relevant data are within the paper and its Supporting Information files.

## Supplementary Materials

The following supporting information can be downloaded at: https://www.mdpi.com/article/10.3390/healthcare10030446/s1, Figure S1: The proportions of prescriptions of each analgesic and its time trends; (a) respiratory diseases, and (b) other diseases.

## References

[1] Moran A.E., Forouzanfar M.H., Roth G.A., Mensah G.A., Ezzati M., Flaxman A., Murray C.J., Naghavi M. (2014). The Global Burden of Ischemic Heart Disease in 1990 and 2010: The Global Burden of Disease 2010 Study. Circulation.

[2] Oliveira G.B., Avezum A., Roever L. (2015). Cardiovascular Disease Burden: Evolving Knowledge of Risk Factors in Myocardial Infarction and Stroke Through Population-Based Research and Perspectives in Global Prevention. Front. Cardiovasc. Med..

[3] Jernberg T., Hasvold P., Henriksson M., Hjelm H., Thuresson M., Janzon M. (2015). Cardiovascular Risk in Post-Myocardial Infarction Patients: Nationwide Real World Data Demonstrate the Importance of a Long-Term Perspective. Eur. Heart J..

[4] Derry S., Moore R.A., Rabbie R. (2016). Topical NSAIDs for Chronic Musculoskeletal Pain in Adults. Cochrane Database Syst. Rev..

[5] Kim S.Y., Chang Y.J., Cho H.M., Hwang Y.W., Moon Y.S. (2015). Non-Steroidal Anti-Inflammatory Drugs for the Common Cold. Cochrane Database Syst. Rev..

[6] Bhala N., Emberson J., Merhi A., Abramson S., Arber N., Baron J.A., Bombardier C., Cannon C., Farkouh M.E., Coxib and traditional NSAID Trialists’ (CNT) Collaboration (2013). Vascular and Upper Gastrointestinal Effects of Non-Steroidal Anti-Inflammatory Drugs: Meta-Analyses of Individual Participant Data from Randomised Trials. Lancet.

[7] Mukherjee D., Nissen S.E., Topol E.J. (2001). Risk of Cardiovascular Events Associated with Selective COX-2 Inhibitors. JAMA.

[8] Trelle S., Reichenbach S., Wandel S., Hildebrand P., Tschannen B., Villiger P.M., Egger M., Jüni P. (2011). Cardiovascular Safety of Non-Steroidal Anti-Inflammatory Drugs: Network Meta-Analysis. BMJ.

[9] Anderson J.L., Adams C.D., Antman E.M., Bridges C.R., Califf R.M., Casey D.E., Chavey W.E., Fesmire F.M., Hochman J.S., Levin T.N. (2013). 2012 ACCF/AHA focused update incorporated into the ACCF/AHA 2007 guidelines for the management of patients with unstable angina/non-ST-elevation myocardial infarction: A report of the American College of Cardiology Foundation/American Heart Association Task Force on Practice Guidelines. Circulation.

[10] Schmidt M., Lamberts M., Olsen A.M., Fosbøll E.L., Niessner A., Tamargo J., Rosano G., Agewall S., Kaski J.C., Kjeldsen K. (2016). Cardiovascular Safety of Non-Aspirin Non-Steroidal Anti-Inflammatory Drugs: Review and Position Paper by the Working Group for Cardiovascular Pharmacotherapy of the European Society of Cardiology. Eur. Heart J. Cardiovasc. Pharmacother..

[11] Purja S., Shin H., Lee J.-Y., Kim E. (2021). Is loss of smell an early predictor of COVID-19 severity: A systematic review and meta-analysis. Arch. Pharm. Res..

[12] Kearney P.M., Baigent C., Godwin J., Halls H., Emberson J.R., Patrono C. (2006). Do Selective Cyclo-oxygenase-2 Inhibitors and Traditional Non-Steroidal Anti-Inflammatory Drugs Increase the Risk of Atherothrombosis? Meta-Analysis of Randomised Trials. BMJ.

[13] Schjerning Olsen A.M., Gislason G.H., McGettigan P., Fosbøl E., Sørensen R., Hansen M.L., Køber L., Torp-Pedersen C., Lamberts M. (2015). Association of NSAID Use with Risk of Bleeding and Cardiovascular Events in Patients Receiving Antithrombotic Therapy After Myocardial Infarction. JAMA.

[14] Kang D.O., An H., Park G.U., Yum Y., Park E.J., Park Y., Jang W.Y., Kim W., Choi J.Y., Roh S.Y. (2020). Cardiovascular and Bleeding Risks Associated with Nonsteroidal Anti-Inflammatory Drugs After Myocardial Infarction. J. Am. Coll. Cardiol..

[15] National Health Insurance Service (NHIS) (2017). What Is Customized Health Information Data?. https://nhiss.nhis.or.kr/bd/ab/bdaba032eng.do.

[16] Quan H., Sundararajan V., Halfon P., Fong A., Burnand B., Luthi J.C., Saunders L.D., Beck C.A., Feasby T.E., Ghali W.A. (2005). Coding Algorithms for Defining Comorbidities in ICD-9-CM and ICD-10 Administrative Data. Med. Care.

[17] Paul A.D., Chauhan C.K. (2005). Study of Usage Pattern of Nonsteroidal Anti-Inflammatory Drugs (NSAIDs) Among Different Practice Categories in Indian Clinical Setting. Eur. J. Clin. Pharmacol..

[18] Al-Shidhani A., Al-Rawahi N., Al-Rawahi A., Sathiya Murthi P. (2015). Non-Steroidal Anti-Inflammatory Drugs (NSAIDs) Use in Primary Health Care Centers in A’Seeb, Muscat: A Clinical Audit. Oman Med. J..

[19] Weber K.L., Jevsevar D.S., McGrory B.J. (2016). AAOS Clinical Practice Guideline: Surgical Management of Osteoarthritis of the Knee: Evidence-Based Guideline. J. Am. Acad. Orthop. Surg..

[20] Calderón-Larrañaga A., Poblador-Plou B., González-Rubio F., Gimeno-Feliu L.A., Abad-Díez J.M., Prados-Torres A. (2012). Multimorbidity, Polypharmacy, Referrals, and Adverse Drug Events: Are We Doing Things Well?. Br. J. Gen. Pract..

[21] Kulik A., Bykov K., Choudhry N.K., Bateman B.T. (2015). Non-Steroidal Anti-Inflammatory Drug Administration After Coronary Artery Bypass Surgery: Utilization Persists Despite the Boxed Warning. Pharmacoepidemiol. Drug Saf..

[22] Roberto G., Bartolini C., Rea F., Onder G., Vitale C., Trifirò G., Kirchmayer U., Chinellato A., Lucenteforte E., Corrao G. (2018). NSAIDs Utilization for Musculoskeletal Indications in Elderly Patients with Cerebro/Cardiovascular Disease. Eur. J. Clin. Pharmacol..

[23] Grau M., Montero J.L., Guasch J., Felipe A., Carrasco E., Juliá S. (1991). The Pharmacological Profile of Aceclofenac, a New Nonsteroidal Antiinflammatory and Analgesic Drug. Agents Actions Suppl..

[24] Kim E., Ihm C., Kang W. (2016). Modeling of Aceclofenac Metabolism to Major Metabolites in Healthy Volunteers. Drug Metab. Pharmacokinet..

[25] Sondergaard K.B., Gislason G. (2017). NSAIDs and Cardiac Arrest: Non-Steroidal Anti-Inflammatory Drug Use Is Associated with Increased Risk of Out-of-Hospital Cardiac Arrest: A Nationwide Case-Time-Control Study. Eur. Heart J..

[26] Kanaki A.R., Patil R.S., Santoshkumar J., Mala R.D. (2013). Comparative Study of Safety, Efficacy, and Tolerability of Aceclofenac Versus Diclofenac in Osteoarthritis Patients. J. Evol. Med. Dent. Sci..

[27] Helin-Salmivaara A., Virtanen A., Vesalainen R., Grönroos J.M., Klaukka T., Idänpään-Heikkilä J.E., Huupponen R. (2006). NSAID Use and the Risk of Hospitalization for First Myocardial Infarction in the General Population: A Nationwide Case-Control Study from Finland. Eur. Heart J..

[28] Kanda A., Ebihara S., Takahashi H., Sasaki H. (2003). Loxoprofen Sodium Suppresses Mouse Tumor Growth by Inhibiting Vascular Endothelial Growth Factor. Acta Oncol..

[29] Misaka E., Yamaguchi T., Iizuka Y., Kamoshida K., Kojima T., Kobayashi K., Endo Y., Misawa Y., Lobayashi S., Tanaka K. (1981). Anti-Inflammatory, Analgesic and Antipyretic Activities of Sodium 2-[4-(2-oxocyclopentan-1-ylmethyl) Phenyl] Propionate Dihydrate (CS−600). Pharmacometrics.

[30] Sakamoto C., Sugano K., Ota S., Sakaki N., Takahashi S., Yoshida Y., Tsukui T., Osawa H., Sakurai Y., Yoshino J. (2006). Case-Control Study on the Association of Upper Gastrointestinal Bleeding and Nonsteroidal Anti-Inflammatory Drugs in Japan. Eur. J. Clin. Pharmacol..

[31] Hwang J.H., Kim D.S., Lee S.I., Hwang J.I. (2007). Relationship Between Physician Characteristics and Their Injection Use in Korea. Int. J. Qual. Health Care.

[32] Lee I.H., Park S., Lee E.K. (2014). Sociodemographic Factors Influencing the Use of Injections in South Korean Outpatient Care. Pharmacoepidemiol. Drug Saf..

[33] Statistics Korea (2017). Statistics on Prescription Rate of Antibiotics and Injections. Statistics Korea. http://www.index.go.kr/potal/main/EachDtlPageDetail.do?idx_cd=1449.

[34] Lee H., Kim E. (2020). Repositioning medication for cardiovascular and cerebrovascular disease to delay the onset and prevent progression of Alzheimer’s disease. Arch Pharm. Res..

