# Supplementary material for: Trends in Ambulatory Analgesic Usage after Myocardial Infarction: A Nationwide Cross-Sectional Study of Real-World Data
Source: Healthcare (Basel). 2022 Feb 26;10(3):446. doi: 10.3390/healthcare10030446 (PMC8956017; doi:10.3390/healthcare10030446)
Supplement: Supplementary file 1 [file healthcare-10-00446-s001.zip › healthcare-1565120-supplementary.pdf]

Supplementary

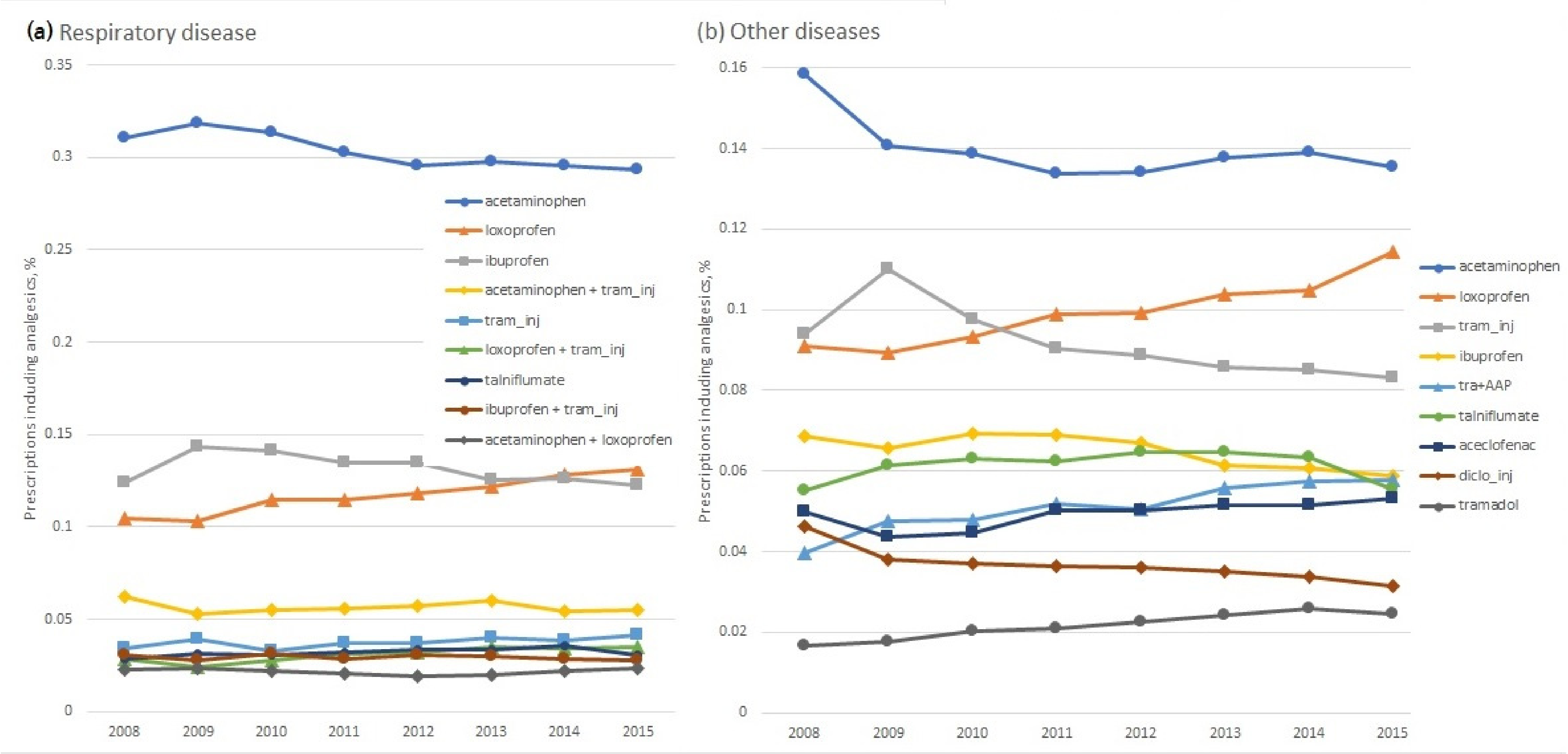

Figure S1 The proportions of prescriptions of each analgesic and its time trends; (a) Respiratory diseases, and (b) other diseases
